# Cancer Vaccines Co-Targeting HER2/Neu and IGF1R

**DOI:** 10.3390/cancers11040517

**Published:** 2019-04-11

**Authors:** Carla De Giovanni, Lorena Landuzzi, Arianna Palladini, Marianna Lucia Ianzano, Giordano Nicoletti, Francesca Ruzzi, Augusto Amici, Stefania Croci, Patrizia Nanni, Pier-Luigi Lollini

**Affiliations:** 1 Laboratory of Immunology and Biology of Metastasis, Department of Experimental, Diagnostic and Specialty Medicine, University of Bologna, Viale Filopanti 22, I-40126 Bologna, Italy; carla.degiovanni@unibo.it (C.D.G.); arianna.palladini@unibo.it (A.P.); marianna.ianzano@unibo.it (M.L.I.); francesca.ruzzi2@unibo.it (F.R.); pierluigi.lollini@unibo.it (P.-L.L.); 2 Laboratory of Experimental Oncology, IRCCS Istituto Ortopedico Rizzoli, I-40136 Bologna, Italy; lorena.landuzzi@ior.it (L.L.); giordano.nicoletti@unibo.it (G.N.); 3 Department of Bioscience and Biotechnology, University of Camerino, I-62032 Camerino, Italy; augusto.amici@unicam.it; 4 Unit of Clinical Immunology, Allergy and Advanced Biotechnologies, Azienda Unità Sanitaria Locale-IRCCS di Reggio Emilia, I-42122 Reggio Emilia; stefania.croci@ausl.re.it

**Keywords:** cancer vaccines, HER2/neu, IGF1R, muscle neoplasms, DNA vaccines

## Abstract

(1) Background: Human epidermal growth factor receptor 2 (HER2)/neu-driven carcinogenesis is delayed by preventive vaccines able to elicit autochthonous antibodies against HER2/neu. Since cooperation between different receptor tyrosine kinases (RTKs) can occur in human as well as in experimental tumors, we investigated the set-up of DNA and cell vaccines to elicit an antibody response co-targeting two RTKs: HER2/neu and the Insulin-like Growth Factor Receptor-1 (IGF1R). (2) Methods: Plasmid vectors carrying the murine optimized IGF1R sequence or the human IGF1R isoform were used as electroporated DNA vaccines. IGF1R plasmids were transfected in allogeneic HER2/neu-positive IL12-producing murine cancer cells to obtain adjuvanted cell vaccines co-expressing HER2/neu and IGF1R. Vaccination was administered in the preneoplastic stage to mice prone to develop HER2/neu-driven, IGF1R-dependent rhabdomyosarcoma. (3) Results: Electroporated DNA vaccines for murine IGF1R did not elicit anti-mIGF1R antibodies, even when combined with Treg-depletion and/or IL12, while DNA vaccines carrying the human IGF1R elicited antibodies recognizing only the human IGF1R isoform. Cell vaccines co-expressing HER2/neu and murine or human IGF1R succeeded in eliciting antibodies recognizing the murine IGF1R isoform. Cell vaccines co-targeting HER2/neu and murine IGF1R induced the highest level of anti-IGF1R antibodies and nearly significantly delayed the onset of spontaneous rhabdomyosarcomas. (4) Conclusions: Multi-engineered adjuvanted cancer cell vaccines can break the tolerance towards a highly tolerized RTK, such as IGF1R. Cell vaccines co-targeting HER2/neu and IGF1R elicited low levels of specific antibodies that slightly delayed onset of HER2/neu-driven, IGF1R-dependent tumors.

## 1. Introduction

Antibodies against the products of receptor tyrosine kinase (RTK) driver oncogenes can impair cancer growth and progression. Passively administered antibodies are successfully included in cancer therapy protocols against targets as HER2 and other RTKs to which tumors can be addicted [1].

Passive immune approaches could have some constraints (for example the need for continuous administration over potentially long-time periods) that could be overcome by active immunization. Active cancer immunoprevention against non-viral targets has, in fact, been known in the literature for several years [2,3,4]. HER2/neu-transgenic mammary carcinogenesis was prevented by HER2/neu-targeted active immune strategies [5], with antibodies and cytokines the main effectors of cancer prevention [6,7], with no reported side effects. Clinical trials for immune-mediated secondary and tertiary prevention of HER2 cancers have also been launched [8,9,10].

HER2 driving oncogene can interact with other RTKs. Insulin-like Growth Factor Receptor-1 (IGF1R) can synergize with HER2 in the induction of invasive potential [11] and is involved in resistance to anti-HER2 treatment [12]. IGF1R is not the main driver of tumor development but is required for transformation and growth [13]. IGF1R plays a role in the growth of different cancer types [14,15,16,17] and immune targeting by specific antibodies or inhibitors has been studied at a clinical level, with limited activity [18,19]. Due to crosstalk and physical interaction between HER2 and IGF1R, combined approaches to co-target both RTKs with antibodies or inhibitors have been suggested [20]. However, an active immune co-targeting of HER2 and IGF1R has never been addressed.

In this paper, in order to set up vaccines co-targeting HER2/neu and IGF1R, we first tried to elicit an active immune response against IGF1R with both DNA and cell vaccines. To date, there have been only a few attempts to obtain an active immune response against IGF1R in a mammary cancer model [21]. We studied the immune prevention of cell vaccines co-targeting HER2/neu and IGF1R to delay the onset of spontaneous HER2/neu-driven, IGF1R-dependent murine tumors. As a model, we chose male BALB-p53Neu mice (p53 knockout, HER2/neu-transgenic BALB/c), which spontaneously develop pelvic rhabdomyosarcoma and salivary carcinoma at an early age [22]. Both tumor types are driven by HER2. In BALB-p53Neu rhabdomyosarcoma, as well as in human rhabdomyosarcoma [16,23], IGF1R-dependence also contributes to tumor growth, as proven by the expression of IGF1R and concomitant autocrine production of IGF2 [22], and by the growth inhibition obtained with anti-IGF1R targeted antibodies [15], small inhibitors, and siRNA or IGF2-directed immune targeting [24].

## 2. Results

### 2.1. DNA Vaccines Against IGF1R

To vaccinate against IGF1R, we chose as DNA vaccines two expression plasmid vectors carrying murine or human isoforms of IGF1R (mIGF1R and hIGF1R, respectively). Concerning mIGF1R plasmid, to obtain a better transduced expression, an optimized mIGF1R sequence was inserted in the expression vector pcDNA3.1, obtaining the pmIGF1Ropt plasmid. Both mIGF1Ropt and hIGF1R plasmids were able to confer on transfected cells the membrane expression of the corresponding IGF1R isoform (see below).

**Figure 1:**
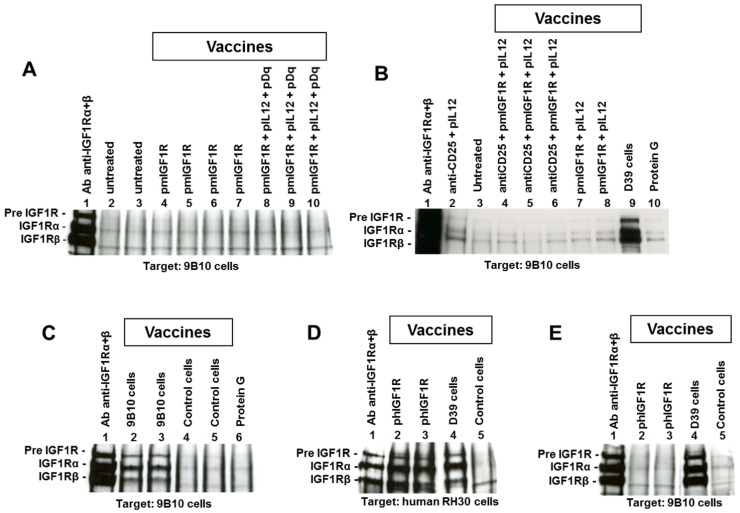
Immunoprecipitation and Western blot analysis of sera to evaluate the induction of antibodies recognizing murine or human Insulin-like Growth Factor Receptor-1 (IGF1R). In each panel sera used for immunoprecipitation are reported over each lane, while tumor cell lysate (target) is reported under each panel. For details, see the Materials and Methods section. (**A**) lane 1, positive control; lanes 2 to 3, sera from untreated BALB/c mice; lanes 4 to 7, sera from BALB/c mice after four vaccinations with pmIGF1Ropt plasmid; lanes 8 to 10, sera from BALB/c mice after four vaccinations with a combination of plasmids (pmIGF1Ropt, pIL12 and pDq). (**B**) lane 1, positive control; lane 2, serum from BALB-p53Neu pretreated with anti-CD25 and vaccinated with pIL12; lane 3, untreated; lanes 4 to 6, sera from BALB-p53Neu mice pretreated with anti-CD25 and vaccinated with pmIGF1Ropt and pIL12 plasmids; lanes 7 to 8, sera from BALB-p53Neu after three vaccinations with pmIGF1Ropt and pIL12 plasmids; lane 9, serum from BALB-p53Neu vaccinated with hIGF1R-expressing D39 cell vaccine (three vaccination cycles); lane 10, protein G alone (negative control). (**C**) lane 1, positive control; lanes 2 to 3, sera from BALB/c mice vaccinated with mIGF1R-expressing 9B10 cells (three cycles); lanes 4 to 5, sera from mice vaccinated with #20 control cells (three cycles); lane 6, protein G alone (negative control). (**D**,**E**) The same sera were used to immunoprecipitate either hIGF1R (**D**) or mIGF1R (**E**) as follows: Lane 1, positive control; lanes 2 to 3, sera from BALB/c mice after four vaccinations with p-hIGF1R plasmid; lane 4, serum from BALB/c mouse after three vaccination cycles with hIGF1R-expressing D39 cells; lane 5, serum from mouse vaccinated with #20 control cells (three cycles).

DNA vaccination against mIGF1R or hIGF1R was performed coupled with electroporation with the purpose to elicit an antibody response recognizing mIGF1R. We conducted this using a protocol we previously set up, that was able to break tolerance of HER2/neu transgenic mice inducing a good antibody response against HER2/neu [25]. Electroporation is considered an adjuvant *per se* [26]. The DNA vaccine carrying the human gene isoform (hIGF1R) takes advantage of a possible adjuvant effect of the non-self, even though it is a highly homologous molecule [27]. No induction of anti-mIGF1R antibodies was observed after electroporated DNA vaccine, either with mIGF1R or hIGF1R isoforms (see Figure 1A, lanes 4 to 7 and Figure 1E, lanes 2 to 3, respectively). DNA vaccine for hIGF1R was able to elicit antibodies against the human isoform hIGF1R (Figure 1D, lanes 2 to 3), as well as DNA vaccination against rat HER2/neu, which was chosen as a positive control for the vaccinal procedure [25], and, as expected, gave a strong antibody response against rat HER2/neu.

We investigated other adjuvant stimuli combined with DNA vaccines, such as combinations with IL12 and allogeneic histocompatibility (H-2D^q^)-carrying plasmids (pIL12 and pDq respectively) (Figure 1A, lanes 8 to 10), with pIL12 alone (Figure 1B, lanes 7 to 8) or Treg depleting mice pretreatment (anti-CD25 antibodies) (Figure 1B, lanes 4 to 6), but anti-mIGF1R antibodies were never elicited.

### 2.2. Cell Vaccines Co-Targeting HER2/neu and IGF1R

**Figure 2:**
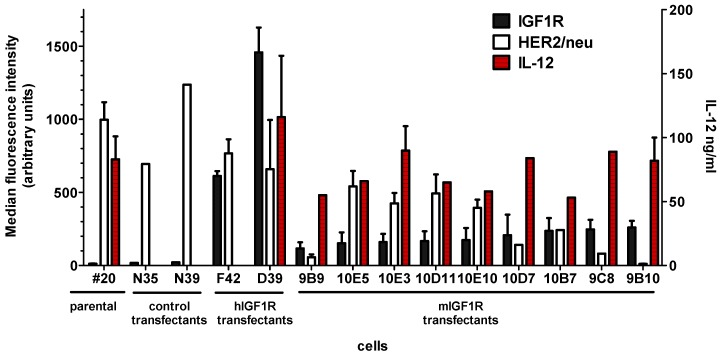
Membrane expression of rat HER2/neu and IGF1R and production of IL12 in #20 cells, in two neomycin-resistant control transfectants, in two hIGF1R-, and in nine mIGF1R-top expressing clones. Mean fluorescence intensity in arbitrary units is reported in the left Y-axis for membrane expression of IGF1R and HER2/neu. Production of IL12 (ng/mL of supernatant) is reported in the right Y-scale. Mean and standard error is shown.

Plasmid vectors pmIGF1Ropt and phIGF1R were transfected in a murine recipient cell line that already expressed transgenic HER2, allogeneic histocompatibility, and transduced murine IL12. The choice of IGF1R transfected clones to be used as cell vaccine was based on the highest reproducible expression level of IGF1R. Clone D39 showed the highest expression of hIGF1R. Clone 9B10 had the highest mIGF1R expression, with a monoclonal profile and a low variability (Table 1). It should be noted that the highest mIGF1R-expressing clones showed a decreased expression of HER2/neu, while production of engineered IL12 was stable (Figure 2).

**Figure 3:**
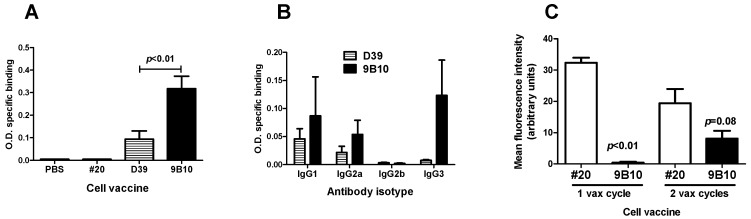
Anti-IGF1R and anti-HER2/neu antibodies elicited by cell vaccines co-targeting HER2/neu and IGF1R. (**A**) Anti-mIGF1R antibodies determined by indirect Elisa assay. Sera were collected from BALB-p53Neu mice after two vaccination cycles and diluted 1:400. Statistical significance (Student’s *t* test) of difference between D39 (*n* = 8) and 9B10 (*n* = 6) is reported in the figure. (**B**) Analysis of antibody isotypes induced by cell vaccines as in Figure 3A (*n* = 3 mice per group). (**C**) Anti-rat HER2/neu antibodies induced in HER2/neu transgenic mice after one or two vaccination cycles and evaluated by indirect immunofluorescence against N10F2.1 target cell (mean fluorescence intensity for rat HER2/neu of 70 arbitrary units). Statistical significance (Student’s *t* test) vs. #20 is reported in the figure (*n* = 3 for each group).

Vaccination with cells expressing mIGF1R or hIGF1R isoform, along with HER2/neu, allogeneic histocompatibility and IL12, succeeded in breaking tolerance against mIGF1R. The mIGF1R-transduced cell vaccine (9B10) gave rise to antibodies recognizing mIGF1R (Figure 1C, lanes 2 to 3). The hIGF1R-transduced cell line D39 elicited antibodies against hIGF1R (Figure 1D, lane 4), which cross-recognize the murine mIGF1R isoform (Figure 1B, lane 9 and Figure 1E, lane 4). Control cell vaccine (#20 recipient cells, expressing HER2, allogeneic H-2D^q^ and IL12, not subjected to transfection with IGF1R gene) did not elicit anti-IGF1R antibodies (Figure 1C, lanes 4 to 5, Figure 1D, lane 5, and Figure 1E, lane 5). Indirect Elisa assay confirmed that IGF1R-transduced and -overexpressing cell vaccines were able to induce antibodies recognizing murine IGF1R (Figure 3A). mIGF1R-expressing 9B10 cell vaccine induced a significantly higher level of antibodies than hIGF1R-expressing D39 cell vaccine. Sera from mice vaccinated with parental #20 cells, as well as sera from non-vaccinated mice, did not show specific binding even at 1:200 dilution (Figure 3A). Predominant isotypes in sera from 9B10 vaccinated mice were IgG1, IgG2a, and IgG3, while no IgG2b were detected (Figure 3B). Such distribution was very close to that previously reported for BALB-p53Neu mice vaccinated with anti-HER2/neu IL12-transduced cell vaccine [25]. Sera from mice vaccinated with hIGF1R-expressing D39 cells lacked the IgG3 isotype.

Data on the comparison of different vaccines allow us to conclude that: (a) DNA vaccine was not able to induce anti-IGF1R antibodies; (b) IGF1R-transduced and -overexpressing adjuvanted cells were able to induce anti-IGF1R antibodies; (c) cells transduced and overexpressing the xenogeneic human IGF1R isoform induced antibodies cross-reacting with the murine IGF1R isoform, but cross-reactivity was less efficient than vaccination with cell transduced with the homologue murine IGF1R isoform. Therefore, the presence of transduced IGF1R in the cell vaccine led to anti-IGF1R antibody responses.

Reasons for the lack of immunogenicity of murine IGF1R DNA vaccines could be a defective B cell activation, likely due to a lack of CD4 T helper induction. Since IL10 was reported to drive anti-IGF1R antibody responses [17], we searched for IL10 production in sera from vaccinated mice, but we did not find any IL10, both after effective cell vaccine and ineffective DNA vaccine. On the contrary, we found IFN-γ release in sera from 9B10 cell vaccinated mice at levels ranging 33 to 164 pg/mL, i.e., in the same magnitude of order as reported previously for anti-HER2 cell vaccines [28]. Sera from DNA vaccinated mice did not show any IFN-γ even when IL12 was included. These data suggest that a Th1 response led to anti-IGF1R antibodies after cell vaccines.

**Figure 4:**
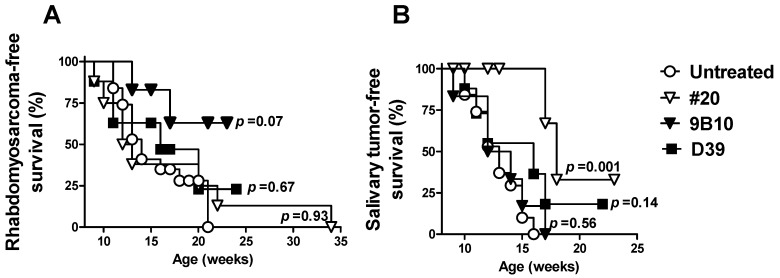
Effect of mIGF1R- or hIGF1R-expressing cell vaccines on the onset of spontaneous rhabdomyosarcoma (**A**) and salivary carcinoma (**B**) in BALB-p53Neu mice. Tumor-free survival (%) is shown. Symbols: Open circles, untreated control mice (*n* = 15); open triangles: #20 cell vaccine (*n* = 8); closed triangles: mIGF1R-expressing 9B10 cell vaccine (*n* = 6); squares: hIGF1R-expressing D39 cell vaccine (*n* = 8). Statistical significance (Mantel–Haenszel test) vs. untreated is reported in the figure. Statistical significance vs. #20 parental cell vaccine is as follows: For rhabdomyosarcomas, D39 *p* = 0.65, and 9B10 *p* = 0.10; for salivary tumors, D39 *p* = 0.12 and 9B10 *p* = 0.006.

To test the preventive ability of vaccines co-targeting HER2/neu and IGF1R, we used p53 knockout, HER2/neu transgenic BALB/c male mice (BALB-p53Neu), which develop at early age HER2/neu-driven, IGF1R-dependent pelvic rhabdomyosarcoma, along with salivary carcinoma [22,29,30]. We previously found that in this model the onset of both rhabdomyosarcoma and salivary carcinoma can be delayed by cell vaccines with a high HER2/neu expression along with adjuvant stimuli [30]. The main effectors of tumor prevention have been shown to be γ-interferon and antibodies [30]. For the present work we chose a cell vaccine with a lower HER2/neu expression (#20), to obtain a suboptimal effect of anti-HER2/neu response, thus allowing us to detect the effects of the anti-IGF1R response. Cell vaccines co-targeting HER2/neu and mIGF1R or hIGF1R (9B10 and D39 cell lines, respectively) were administered to BALB-p53Neu mice starting at an early age and corresponding to preneoplastic stage [22,29] (Figure 4).

A nearly significant delay of rhabdomyosarcoma onset was obtained with the mIGF1R-expressing cell vaccine compared to spontaneous onset or to other cell vaccines (Figure 4A). Such preventive effect by 9B10 cell vaccine, and not by D39, was in agreement with the higher level of anti-mIGF1R antibodies induced (see Figure 3A). The onset of salivary carcinoma in the mice, rapidly leading to death, limited the possibility to detect a further delay in rhabdomyosarcoma onset. In fact, the significant delay in salivary carcinoma onset induced by parental #20 cell vaccine was lost with both IGF1R-expressing cell vaccines (Figure 4B). The cell vaccine expressing mIGF1R, elicited a lower level of anti-HER2/neu antibodies than parental #20 cells; anti-HER2/neu antibodies became detectable only after the second vaccination cycle (Figure 3C). Therefore, reduced preventive efficacy of IGF1R transfectants is likely due to the decreased HER2/neu expression of IGF1R-transduced vaccines that elicited a lower anti-HER2/neu response, since it was reported that a preventive effect correlated with the entity and speed of the anti-HER2/neu response elicited [30].

## 3. Discussion

In this paper we investigated the possibility to exploit immunity with vaccines co-targeting two RTKs, HER2/neu and IGF1R, to delay the onset of tumors driven by HER2/neu and depending on IGF1R.

We previously reported that both DNA and cell vaccines can break tolerance towards HER2/neu, giving rise to antibodies which can prevent HER2/neu-driven carcinogenesis, if elicited at a preneoplastic stage and at sufficiently high levels [25,30,31]. In this paper, we investigated the possibility to set up DNA and cells vaccines against IGF1R to be combined with anti-HER2/neu vaccines for co-targeting of the two RTKs.

DNA vaccines against IGF1R were first investigated, since they could be easily combined with anti-HER2/neu DNA vaccine [25]. To break tolerance towards IGF1R, we exploited different strategies, such as the use of a codon-optimized mIGF1R sequence and the combination with adjuvant stimuli, such as the Th1-driving cytokine IL12, the expression of allogeneic histocompatibility molecule, and a Treg-depleting treatment [6,32]. Since the use of the xenogeneic gene for DNA vaccine has been reported as an adjuvant for tolerance breakage in other systems [33,34], we also used DNA vaccine coding for the human IGF1R isoform. While all the above reported protocols easily induced high-level anti-HER2 antibodies, we did not elicit antibodies recognizing murine IGF1R. Reasons for this difference could be a leaky tolerance to a transgene (HER2), or, alternatively, the importance of the IGF1R system that evolved as a tightly tolerized system [35]. The relationships between immunogenicity and tolerogenicity of IGF1R peptides concerning T-helper epitopes, have been studied in depth [17]. Extracellular and transmembrane domains elicited Th2 responses, with higher frequency in breast cancer patients, while intracellular epitopes induced Th1 immunity in tumor-bearing patients, as well as in healthy volunteers. In our system, anti-IGF1R DNA vaccine, even when adjuvants were present, did not elicit Th1 or Th2 responses, suggesting a low B cell activation, likely due to a lack of CD4 T helper induction. Induction of antibodies was obtained with similar DNA protocols against other non-transgenic RTKs [36].

Cell vaccines overexpressing transduced murine or human IGF1R, along with HER2/neu and adjuvant stimuli (allogenicity and IL12 production) were able to elicit antibodies recognizing murine IGF1R, with evidence of an IFN-γ-mediated Th1 response, partly due to the adjuvant and Th1-polarizing effect of engineered IL-12. However, a phenomenon we reproducibly observed was that selection for IGF1R-overexpressing clones was accompanied by a decreased expression of HER2. The crosstalk and physical interaction between HER2 and IGF1R [20,37] could be involved in this phenomenon. An alternative approach could have used a vaccine containing HER2/neu-expressing cells admixed with IGF1R-expressing cells. We chose to transfect IGF1R in HER2/neu-expressing cells to obtain cells with high expression of both RTKs, essentially for two reasons. The first one is that HER2-positive human cancer cells usually co-express IGF1R [20,37]. The second reason is that the moderate spontaneous expression of IGF1R usually found, could be insufficient to break tolerance against IGF1R.

We then investigated whether cell vaccines co-targeting HER2 and IGF1R could delay the onset of HER2-driven, IGF1R-dependent rhabdomyosarcoma and salivary carcinoma. A slight, nearly significant, delay of rhabdomyosarcoma onset was obtained with cell vaccine co-targeting mIGF1R and HER2, while cell vaccine expressing only HER2 and adjuvants gave superimposable onset compared to the non-vaccinated control group. The almost contemporaneous onset of salivary carcinoma limited the follow-up observation period and could have led to an underestimation of the preventive effect against rhabdomyosarcoma. Salivary tumors were significantly delayed by the cell vaccine expressing only HER2 and adjuvants, but such preventive efficacy against HER2-driven salivary tumors was lost with IGF1R-expressing cell vaccine likely due to the decreased HER2 expression level and to the corresponding lower induction of anti-HER2 antibodies elicited.

In conclusion, cell vaccines overexpressing IGF1R along with other immune stimuli succeeded in breaking the tolerance toward IGF1R, but the obtained immune response only marginally affected tumor onset. This was likely due to the low antibody level elicited.

Highly engineered cancer cells were the most powerful immunogen in the induction of anti-IGF1R antibodies, as well as in the induction of anti-HER2 antibodies in several other systems, including humanized mice [38]. The demonstration that multi-engineered adjuvanted cancer cell vaccines can break the tolerance towards a highly tolerized RTK, such as IGF1R, is a valuable proof of principle.

## 4. Materials and Methods

### 4.1. Cells, Culture Conditions, and Reagents

The following cells were used throughout the study: Neu/H-2q/IL12 clone #20 cells (here indicated as #20), derived from a mammary carcinoma arisen in a FVB female mouse transgenic for rat HER2/neu, and engineered for production of murine IL12 and hygromycin resistance [39]; N10-F2.1 cells, derived from a mammary carcinoma expressing rat HER2/neu arisen in HER2/neu transgenic mice of BALB/c background (our unpublished results); SJ-Rh30 human rhabdomyosarcoma [40]. Cells were routinely grown in adherence in Dulbecco’s Modified Eagle Medium supplemented with 10% to 20% fetal bovine serum.

### 4.2. Plasmids and Transfections

The plasmid pCVNIGF1R, carrying the normal full-length human IGF1R cDNA under the SV40 promoter, was kindly given by Dr. Baserga [41,42]. Murine IGF1R plasmid pmIGF1R-opt derived from pcDNA3.1 plasmid by insertion of a codon-optimized cDNA sequence coding for normal murine IGF1R (Genscript Corporation, Piscatway, NJ, USA) (see Supplementary Materials) under Cytomegalovirus promoter. Plasmid largescale production and purification were performed with EndoFree Plasmid Giga kits (QIAGEN, Valencia, CA, USA). The day after seeding, #20 cells were transfected with 20 µg pCVNIGF1R with Calcium-Phosphate Precipitation (Thermo Fisher Scientific, Rodano, MI, Italy) or 1 µg pmIGF1Ropt and 3 µL of Fugene (Thermo Fisher Scientific), according to the protocols suggested by the manufacturers. Control transfectants were obtained with pcDNA3.1 alone. Cells were cloned and stable neomycin-resistant clones were isolated and selected for high expression of human IGF1R (clone D39) or murine IGF1R (clone 9B10), as evaluated by indirect immunofluorescence and cytofluorometric analysis with FACScan flow cytometer (BD, Mountain View, CA, USA). IGF1R and HER2/neu expression were routinely verified and found stable up to at least 12 in vitro passages (corresponding to about 3 months of continuous culture). Transfectant cells were frozen and aliquots were periodically thawed to perform experiments and cultured in vitro for no more than 10 passages.

### 4.3. Ethics Statement

All animal experiments were performed according to European directive 2010/63/UE and Italian law (DL 26/2014). Experimental protocols were reviewed and approved by the Institutional Animal Care and Use Committee (“Comitato per il Benessere Animale”) of the University of Bologna, and the Italian Ministry of Health with letters n. 71674-x/6, 12511-x/10 and 4783-X/10, (supervisor: Prof. Patrizia Nanni).

### 4.4. Mice

BALB/c p53^+/−^ female mice (BALB/cJ-Trp53tm1Tyj, purchased from The Jackson Laboratory, Bar Harbor, MI, USA) were crossed with BALB/c HER2/neu transgenic male mice, carrying a mutant rat neu oncogene (NeuT) under control of a mammary tumor virus-long terminal repeat promoter, bred in our facilities as described [6]. Mice bearing the p53^+/−^/neu^+/−^ genotype (referred to as BALB-p53Neu) were selected by polymerase chain reaction (PCR) genotyping [22]. Male BALB-p53Neu mice develop salivary gland carcinomas and pelvic rhabdomyosarcoma in urethral tissue proximal to bladder at about 13 to 15 weeks of age [22]. Wild-type BALB/c AnNCrlBR (BALB/c) mice were purchased from Charles River Italy, Calco, Lecco, Italy.

### 4.5. DNA and Cell Vaccinations

DNA vaccination consisted of intramuscular injections of 50 μg of plasmid diluted to a final volume of 40 μL per mouse in final concentrations of 0.9% NaCl and 6 mg/mL polyglutamate. Anesthetized mice received the injection of DNA vaccine (pCVNIGF1R or pmIGF1R-opt) into the tibial muscles (20 μL in each muscle) through a 28-gauge needle syringe. Immediately thereafter, the muscle tissues were subjected to electroporation, consisting of two square wave, 25-ms, 375 V/cm pulses generated with a T830 electroporator (BTX, San Diego, CA, USA). Mice received up to four i.m. injections (at 6, 8, 13, and 15 weeks of age). DNA vaccine pmIGF1R-opt was also coupled with the administration of antibody causing Treg depletion or with other plasmid, such as pIL12-IRES1neo (able to confer expression of murine IL12, here referred to as pIL12) or pDq (able to transfer H-2Dq expression) [25]. Five and four days before the first vaccination, mice received anti-CD25 (PC61) rat monoclonal antibody at the dose of 500 µg/mouse (kind gift from Dr. Ferrini S. Istituto Nazionale per la Ricerca sul Cancro, Genoa, Italy). Cell vaccines consisted of IL12-producing HER2-expressing H-2^q^ murine mammary carcinoma cells stably transfected with murine or human IGF1R (clones 9B10 and D39, respectively). Vaccine cells were proliferation-blocked by treatment with mitomycin C (40 μg/mL Sigma-Aldrich, Milan, Italy) and administered intraperitoneally in 0.4 mL of phosphate-buffered saline (PBS) (Invitrogen, Milan, Italy). Control mice received PBS alone. A vaccination cycle consisted of 4 vaccinations over 2 weeks, followed by 2 weeks of rest [39]. Vaccination cycles were repeated ad libitum. Vaccinated mice were bled via the tail vein and serum samples were stored at −80 °C. Production of antibodies was studied by immunoprecipitation/Western blot, Elisa assay and by indirect immunofluorescence.

### 4.6. Immunoprecipitation/Western Blot and Elisa Assays

Production of antibodies anti-IGF1R was analyzed by immunoprecipitation followed by Western blot. Cells expressing murine IGF1R (cell line 9B10) or human IGF1R (SJ-Rh30) were lysed with 50 mM Tris-HCl (pH 7.5), 1 mM EDTA, 1% Igepal, 0.5% sodium deoxycholate, 0.1% SDS, 10% glycerol, 150 mM NaCl plus phosphatase and protease inhibitors (all reagents were purchased from Sigma, Milan, Italy) for 30 min on ice. Nuclei were removed by centrifugation at 12,000× *g* at 4 °C for 15 min and protein concentration in the supernatants was determined by DC Protein Assay (Bio-Rad, Milan, Italy) using BSA as standard protein and absorbance was measured at 750 nm using an ELISA microreader (Tecan Systems, San Jose, CA, USA). To immunoprecipitate IGF1R we used Dynabeads Protein G (Thermo Fisher Scientific). 10 µL of serum or 1:1 pool of two sera were added to 350 µg of total cellular proteins and incubated overnight at 4 °C. 1 µg of a rabbit polyclonal antibody IGF1Rβ (C-20) plus 1 µg of a rabbit polyclonal antibody IGF1Rα (N-20), (purchased by Santa Cruz Biotechnology Inc, Dallas, TX, USA) added to cellular proteins were used as positive control. Cellular lysate alone was used as negative control (aspecific signal of protein G). 24 h later, 40 µL of beads were added to antigen-antibody complex and incubated at 4 °C with agitation for 1h and 30 min. Immunoprecipitates were collected using a DynaMag magnet. Then pellets were washed with PBS + Tween 20 0.1% buffer (Biorad, Milan, Italy), resuspended in 25 µL of Laemmli Sample Buffer 2× (Tris-HCl 62.5 mM pH 6.8, 25% glycerol, 2% SDS, 0.01% bromophenol blue, Bio-Rad Laboratories, Segrate, MI, Italy) with 5% β-Mercaptoethanol (Sigma) and denatured at 95 °C for 10 min. For Western Blot, immunoprecipitates were separated on an 8% polyacrylamide gel, then transferred to polyvinylidene difluoride membranes (Bio-Rad). After blocking with PBS containing 0.1% Tween 20 plus 5% non-fat dry milk for two hours at room temperature, membranes were incubated overnight at 4 °C with a mix of primary antibodies diluted in blocking buffer: Anti-IGF1R rabbit polyclonal antibody (C-20) 0.5 μg/mL plus rabbit polyclonal antibody IGF1Rα (N-20) 0.5 μg/mL. Protein presence was detected through the incubation with the horseradish peroxidase-labeled secondary antibodies (Santa Cruz Biotechnology) revealed by chemiluminescent reaction (Clarity Western ECL Substrate, Biorad, Milan, Italy).

Elisa assay for anti-mIGF1R antibodies was performed in MicroWell^TM^ Maxisorp^TM^ NUNC microplates after overnight coating with recombinant murine IGF1R (R&D Systems, Minneapolis, MN, USA) at 40 ng/well in carbonate buffer [17]. Sera were diluted 1:200 to 1:400 in assay buffer (4% bovine serum albumin in phosphate-buffered saline) and Elisa was performed following the protocol reported previously [43]. Since no anti-mIGF1R mouse antibody was available to set up a standard curve, a semiquantitative evaluation was done expressing results as O.D. specific binding, calculated as “O.D. of mIGF1R-coated wells—O.D. of buffer-coated wells”. Isotype subclass analysis was carried out in Elisa assays with secondary biotin-labeled anti-mouse IgG1 (clone LO-MG1-2), IgG2a (clone LO-MG2a-2), IgG2b (clone LO-MG2b-2), and IgG3 (clone LO-MG3-7), all purchased from AbD Serotec, Bio-Rad Laboratories. Samples were then incubated with alkaline phosphatase-conjugated Streptavidin (AbD Serotec), developed with p-nitrophenyl phosphate (Sigma-Aldrich) and specific binding calculated as above.

Elisa assays for serum murine cytokines IFN-γ and IL10 were purchased from Affymetrix eBiosciences and R&D Systems, respectively. Sera were collected two weeks after the second DNA vaccination (10 to 11 weeks of age) or two weeks after the second cycle of cell vaccination (12 to 13 weeks of age).

### 4.7. Indirect Immunofluorescence

Anti-HER2/neu antibody level was studied by indirect immunofluorescence of rat HER2/neu-expressing N10F2.1 cells (with BALB/c background). N10F2.1 cells were incubated with sera at a 1:65 dilution for 30 min in ice, and then washed and incubated with an Alexa Fluor 488 F(ab’)2 fragment of goat anti-mouse IgG (H-L) chains antibody (Invitrogen Molecular Probes, Eugene, OR, USA). Cytofluorometric analysis was performed (Sysmex-Partec Cy Flow Space, Carate Brianza, MB, Italy). Rat HER2/neu expression of N10F2.1 target cells was evaluated in parallel tests with anti-c-ErbB2/Neu mouse monoclonal antibody (clone 7.16.4; Oncogene Research Products, Boston, MA, USA).

### 4.8. Statistical Analysis

Differences in tumor-free survival curves were analyzed by the Mantel–Haenszel test. Antibody levels were compared by the Student’s *t* test or nonparametric Wilcoxon test.

## 5. Conclusions

Multi-engineered adjuvanted cancer cell vaccines can break the tolerance towards a highly tolerized RTK, such as IGF1R. Cell vaccines co-targeting HER2/neu and IGF1R elicited low levels of specific antibodies that partly delayed onset of HER2-driven, IGF1R-dependent tumors.

## Figures and Tables

**Table 1:** IGF1R-expressing cells and control transfectants.

Cells	Vector	Resistance Gene	IGF1R Gene	IGF1R Membrane Expression (mfi)	HER2/neu Membrane Expression (mfi)	IL12 in 72 h Culture (10^6^ Cells Seeded) ng/mL
#20	-	-	-	12 ± 2	997 ± 120	88 ± 29
#20/N	pcDNA3.1	neo	-	10 ± 4	966 ± 271	nd
D39	pCVNIGF1R	neo	Human	1458 ± 170	659 ± 337	116 ± 48
9B10	pmIGF1Ropt	neo	Murine (optimized)	260 ± 45	11 ± 2	83 ± 18

mfi = median fluorescence intensity; nd = not done.

## Supplementary Materials

The following are available online at https://www.mdpi.com/2072-6694/11/4/517/s1, Supplementary Materials: Optimized sequence of the murine IGF1R gene; Optimized Sequence: Length: 4316, GC%: 50.65, Minimum Free Energy: 1519.80.
