# Supplementary material for: Cancer Vaccines Co-Targeting HER2/Neu and IGF1R
Source: Cancers (Basel). 2019 Apr 11;11(4):517. doi: 10.3390/cancers11040517 (PMC6520928; doi:10.3390/cancers11040517)
Supplement: Supplementary file 1 [file cancers-11-00517-s001.pdf]

Supplementary Materials:

Cancer Vaccines Co-Targeting HER2/Neu and IGF1R

Carla De Giovanni, Lorena Landuzzi, Arianna Palladini, Marianna Lucia Ianzano, Giordano Nicoletti, Francesca Ruzzi, Augusto Amici, Stefania Croci, Patrizia Nanni and Pier-Luigi Lollini

Optimized sequence of the murine IGF1R gene

Optimized Sequence: Length: 4316, GC%: 50.65, Minimum Free Energy: 1519.80

AGTGAGGACTGAGTTGGAGACTTTTTTTTTCTTTTTTTCTTTTCTTTTTTTTTTTTTTTCTAT
TTTTGAGAAAAGGGAATTTCGTCCCAAATAAAAGGAGCCACCATGAAGAGTGGAAGTGGTG
GAGGTAGCCCTACCAGTCTCTGGGGGCTGGTGTTTCTGTCAGCCGCTCTCAGCCTTTGGCCCA
CCTCCGGGGAGATCTGTGGCCCCGGAATCGATATCAGAAACGATTACCAGCAGCTGAAAAG
ACTGGAAAATTGCACCGTCATTGAGGGCTTCTTGCACATCCTGCTGATTTCCAAAGCTGAAG
ACTATAGGTCATATAGGTTCCCTAAACTGACCGTGATAACAGAGTATCTGCTTCTGTTTCGCG
TGGCCGGCCTGGAATCCCTGGGCGATCTGTTTCCTAATCTCACAGTGATCAGAGGCTGGAAG
CTGTTCTACAACTACGCTCTGGTTATTTTCGAAATGACTAACCTGAAGGACATAGGGCTCTAC
AACCTCAGGAACATCACCCGGGGCGCTATTCGGATCGAGAAAAATGCAGATCTGTGCTATCT
GTCTACCATTGATTGGTCACTGATTCTGGATGCTGTCTCTAATAATTACATCGTGGGGAATAA
GCCCCCTAAAGAATGCGGGGACCTTTGTCCTGGCACACTCGAGGAGAAACCAATGTGTGAA
AAGACGACCATTAACAATGAGTATAACTATCGCTGTTGGACTACAAATAGATGCCAGAAGA
TGTGCCCTTCTGTGTGTGGGAAGCGCGCATGTACCGAAAATAATGAGTGTTGTCACCCCGAA
TGTCTTGGGTCCTGTCACACACCTGACGACAATACTACCTGTGTCGCCTGTAGGCATTATTAT
TATAAAGGAGTTTGTGTTCCAGCCTGCCCCCCAGGGACTTATCGCTTTGAAGGCTGGCGGTGC
GTGGACAGAGACTTTTGCGCTAACATTCCAAACGCCGAGTCCAGTGACTCCGATGGTTTCGT
CATTCATGACGATGAATGTATGCAGGAATGCCCTAGCGGGTTTATACGGAACTCTACCCAGT
CTATGTACTGTATTCCATGTGAAGGACCCTGTCCCAAAGTTTGTGGGGATGAAGAGAAGAAG
ACAAAGACCATTGACAGCGTGACCTCAGCCCAGATGTTGCAGGGATGTACGATTCTGAAGG
GAAATCTCCTCATCAACATTAGAAGGGGGAATAATATCGCAAGTGAACTGGAAAACTTTAT
GGGACTGATAGAGGTCGTGACCGGGTATGTGAAGATTCGGCATAGCCACGCCCTTGTCTCTC
TCAGCTTTCTCAAAAATCTGAGACTGATCCTGGGCGAGGAACAGCTTGAAGGTAACTACTCC
TTTTACGTCCTCGACAACCAGAATCTGCAACAGCTGTGGGACTGGAACCACCGTAACCTCAC
CGTGCGATCCGGAAAAATGTATTTTGCATTCAATCCAAAGCTGTGCGTTTCCGAGATCTACCG
GATGGAAGAGGTCACGGGAACGAAGGGAAGACAGTCAAAAGGAGATATCAACACTAGGA
ATAATGGAGAGCGGGCTTCCTGCGAAAGTGACGTGCTCAGATTCACCTCCACCACCACTTGG
AAAAATCGAATTATCATTACGTGGCACCGTTATCGACCCCCTGATTATAGAGACCTGATCAG
TTTCACCGTGTACTACAAGGAGGCTCCCTTTAAAAACGTTACAGAATACGACGGTCAGGATG
CCTGCGGATCCAATTCATGGAATATGGTGGACGTGGATCTGCCTCCAAACAAAGAAGGGGA
GCCAGGCATTCTTCTTCACGGCCTCAAACCGTGGACCCAGTATGCCGTCTATGTGAAGGCTGT
GACCCTGACAATGGTCGAGAATGATCACATCCGGGGAGCCAAGTCTGAAATTCTGTACATTA
GAACTAACGCTAGTGTCCCTAGTATCCCTCTCGACGTGCTGTCAGCCTCCAACTCTTCCAGCC
AGCTGATCGTCAAATGGAACCCCCCGACTCTTCCGAACGGGAATCTGTCCTATTACATCGTG
CGCTGGCAGAGGCAACCACAGGATGGGTATCTGTACCGGCACAACTATTGTAGTAAGGATA
AAATTCCAATTAGGAAGTATGCAGATGGGACCATTGACGTGGAGGAGGTTACTGAGAACCC
CAAGACAGAGGTTTGTGGAGGGGATAAGGGACCGTGTTGTGCCTGCCCCAAGACAGAGGCC
GAAAAACAGGCCGAAAAGGAGGAAGCTGAGTATCGCAAGGTCTTCGAGAACTTCCTTCATA
ACTCTATCTTTGTCCCCAGGCCTGAACGTAGACGGAGAGATGTGATGCAAGTGGCCAATACG
ACCATGTCTAGTAGGAGTCGCAATACTACCGTCGCCGACACATATAACATCACAGACCCGG
AGGAGTTCGAGACTGAATATCCCTTCTTTGAGAGCAGGGTGGACAATAAGGAAAGAACAGT
GATCAGCAACCTGCGACCGTTCACACTGTACAGGATCGATATTCACTCCTGTAACCACGAAG
CCGAGAAGCTGGGCTGCAGTGCAAGTAACTTTGTCTTCGCCCGAACTATGCCCGCAGAGGGT

GCCGACGATATTCCCGGCCCCGTTACCTGGGAACCGAGGCCCGAGAACTCTATCTTCCTCAA
ATGGCCAGAGCCCGAGAACCCTAATGGTCTTATCCTGATGTATGAGATCAAGTACGGCAGCC
AGGTGGAAGACCAGAGAGAATGTGTCTCCAGGCAAGAATACAGGAAGTATGGTGGGGCAA
AACTGAACCGGTTGAATCCGGGAAATTATACTGCCAGGATCCAAGCCACTTCTCTCAGTGGC
AATGGGTCCTGGACAGATCCTGTGTTCTTCTACGTGCCAGCCAAGACAACCTATGAAAACTT
TATGCACCTCATTATTGCCCTCCCAGTTGCTATCCTCCTTATCGTGGGTGGACTGGTCATCATG
CTTTATGTCTTTCACCGGAAGCGCAACAATAGTAGACTGGGTAACGGCGTCCTGTATGCTAGT
GTCAATCCGGAGTACTTTTCAGCAGCTGACGTCTACGTGCCTGATGAGTGGGAAGTGGCCCG
CGAAAAAATCACCATGAACAGAGAACTGGGCCAGGGCAGCTTTGGAATGGTCTATGAGGGT
GTCGCTAAAGGAGTCGTGAAGGACGAGCCAGAAACTCGGGTGGCCATTAAGACTGTGAATG
AAGCCGCTTCTATGAGGGAGAGAATCGAATTTCTCAACGAGGCCAGCGTCATGAAGGAGTT
CAATTGTCACCATGTTGTGCGGCTCCTTGGCGTGGTCAGTCAGGGCCAGCCGACACTTGTTAT
CATGGAGCTGATGACCCGTGGGGACCTCAAGTCCTACCTGCGATCTCTCCGACCGGAAGTTG
AGCAGAACAATCTGGTCCTGATACCCCCTAGTCTGAGCAAGATGATTCAGATGGCCGGAGA
AATTGCCGATGGAATGGCCTATCTGAACGCCAACAAATTCGTGCATAGGGATCTTGCAGCTC
GAAATTGCATGGTGGCTGAGGATTTCACTGTGAAGATCGGAGACTTTGGCATGACAAGGGAC
ATCTACGAAACAGACTACTATCGCAAGGGAGGCAAAGGCCTGCTTCCAGTTCGGTGGATGA
GCCCCGAGTCACTGAAAGATGGTGTCTTCACCACCCACTCAGATGTCTGGAGCTTTGGAGTG
GTCCTGTGGGAAATCGCCACCCTGGCTGAACAGCCATACCAGGGCTTGTCCAACGAGCAGGT
TCTGCGATTCGTCATGGAGGGGGGCCTCTTGGACAAGCCTGACAACTGCCCCGATATGCTGT
TCGAACTGATGAGAATGTGCTGGCAATACAACCCCAAGATGCGACCGTCTTTCCTGGAGATA
ATTGGGTCTATCAAGGATGAGATGGAACCCAGCTTCCAGGAGGTGAGCTTCTATTATAGCGA
GGAAAACAAACCTCCCGAGCCAGAGGAACTGGAGATGGAGCCAGAGAATATGGAGTCAGT
TCCACTCGACCCTAGCGCCAGCAGTGCCTCACTCCCTCTCCCTGAACGACACAGTGGGCACA
AGGCAGAGAACGGCCCAGGTCCGGGGGTTCTTGTGCTTAGAGCCTCTTTTGATGAGCGGCAG
CCATATGCACACATGAATGGGGGGAGAGCCAACGAGCGCGCTCTGCCTCTGCCACAGAGCT
CTACCTGCTGATCCTCGGACACACCGAAGCACGCGCCAACAGTAACGTGTGTGCGCCCACTC
GGTGGGCGGGGGGGCGGGGAGGGGAGAGCAGGTTGTAACAATCTATTCA

© 2019 by the authors. Licensee MDPI, Basel, Switzerland. This article is an open access article distributed under the terms and conditions of the Creative Commons Attribution (CC BY) license (http://creativecommons.org/licenses/by/4.0/).
